# Supplementary material for: Fabrication and Characterization of Polylactic Acid Electrospun Wound Dressing Modified with Polyethylene Glycol, Rosmarinic Acid and Graphite Oxide
Source: Nanomaterials (Basel). 2023 Jul 3;13(13):2000. doi: 10.3390/nano13132000 (PMC10343514; doi:10.3390/nano13132000)
Supplement: Supplementary file 1 [file nanomaterials-13-02000-s001.zip › nanomaterials-2474028-supplementary.pdf]

Supplementary Materials

Fabrication and Characterization of Polylactic Acid Electrospun Wound Dressing Modified with Polyethylene Glycol, Rosmarinic Acid and Graphite Oxide

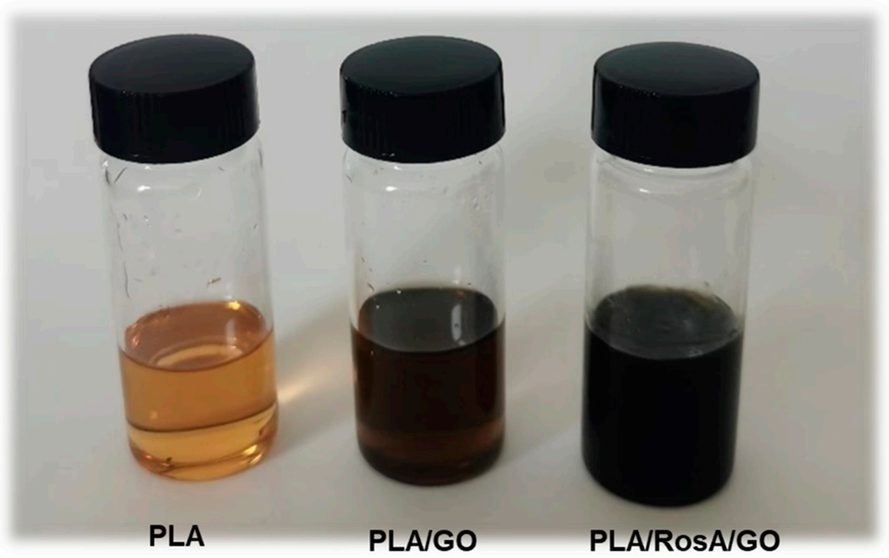

Figure.S1 The photos of solution of PLA, PLA/GO and PLA/RosA/GO in mixed solvents of DCM and DMF (8:2, *v/v*)

Table S1 Thermal weight loss based TG curves

Samples	5% weight loss	10% weight loss
PLA	288	310
PLA/RosA/GO	310	324
PLA/PEG/RosA/GO	315	328

Table S2 Healing rates after treatment in different treatment groups

	Healing rate		
Time (Days)	Control	PLA/RosA/GO	PLA/PEG/RosA/GO
1	0.00%	0.00%	0.00%
3	20.49%	22.74%	34.17%
5	37.11%	48.82%	53.09%
7	57.01%	69.54%	70.13%
9	76.66%	88.23%	87.96%
11	84.57%	95.26%	94.17%
